# Rheumatoid ulcer in a case of rheumatoid arthritis with pulmonary tuberculosis

**DOI:** 10.11604/pamj.2024.48.147.44304

**Published:** 2024-08-02

**Authors:** Souvik Sarkar, Poonam Patil

**Affiliations:** 1 Department of Respiratory Medicine, Datta Meghe Institute of Higher Education and Research, Wardha, Maharashtra, India

**Keywords:** Rheumatoid arthritis, non-healing ulcer, tuberculosis

## Image in medicine

A 49-year-old male, non-diabetic and non-hypertensive, diagnosed with rheumatoid arthritis, previously treated with methotrexate and leflunomide, presented with a painful lesion on his right lower limb, fever with chills, and a cough with expectoration for 2 months. He had stopped his rheumatoid arthritis medication 6 months prior. On examination, he was emaciated and febrile, had a pulse rate of 108 bpm, blood pressure of 80/50 mmHg, and oxygen saturation of 88% on ambient air. The lesion was a 3 cm ulcer on the right knee with an undermined edge, regular margin, and healthy granulation tissue without discharge. Cultures from the ulcer showed no growth for bacteria or tuberculosis. Rheumatoid factor and anti-cyclic citrullinated peptide (anti-CCP) were elevated. Chest computed tomography (CT) revealed a cavity in the left upper lobe and fibrotic changes in the bilateral lower lobes. Sputum examination and cartridge-based nucleic acid amplification test (CBNAAT) detected acid-fast bacilli without rifampicin resistance. The patient was started on oral anti-tubercular treatment (isoniazid, rifampicin, pyrazinamide, ethambutol) and received daily ulcer dressings. A rheumatologist recommended starting oral steroids and would review after completing the intensive phase of anti-tubercular treatment.

**Figure 1:**
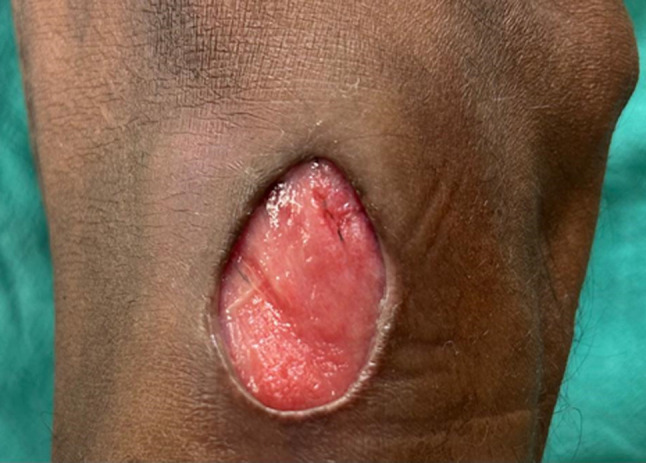
circular ulcer of 3-centimeter diameter located on the lateral aspect of the right knee, with undermined edges and pink granulation tissue at the floor, suggestive of rheumatic ulcer

